# Embeddable Chloride Sensor for Monitoring Chloride Penetration into Cement Mortar

**DOI:** 10.3390/s24072149

**Published:** 2024-03-27

**Authors:** Min Zhang, Hua Fu, Li Tian, Zhenxing Du, Penggang Wang

**Affiliations:** School of Civil Engineering, Qingdao University of Technology, Qingdao 266033, China; zhangmin09588@163.com (M.Z.); fs215379@163.com (H.F.); tlsxf@163.com (L.T.); wangpenggang007@163.com (P.W.)

**Keywords:** Ag/AgCl electrode, Mn/MnO_2_ electrode, chloride sensor, in situ monitoring

## Abstract

A composite solid chloride sensor consisting of two single sensors, i.e., Ag/AgCl working electrode and Mn/MnO_2_ reference electrode, was developed. The Ag/AgCl electrode was prepared by the anodic polarization method, while the Mn/MnO_2_ reference electrode was prepared using the powder compaction technique. Then, the electrochemical performances such as stability, reproducibility, and sensitivity of the composite and single sensors were investigated in a saturated Ca(OH)_2_ solution and mortar specimen. A current density of 0.5 mA/cm^2^ and polarization time of 2.5 h were the optimal preparation parameters of the Ag/AgCl selective electrode. The Ag/AgCl selective electrode showed a linear potential response with the logarithm of chloride ion content in solution and had good stability, reproducibility, and anti-polarization performances. In addition, the Mn/MnO_2_ electrode exhibited potential stability after being activated in an alkaline solution for 60 days. The composite sensor demonstrated exceptional sensitivity to the Cl^−^ content, boasting a slope of approximately 51.1 mV/decade, and showcased excellent stability in both solution and mortar specimens. In every measurement, the time needed for the potential of a composite sensor to become stable was less than 30 s. The sensor enables non-destructive in situ monitoring of the chloride ion content in cement mortar, thus realizing early warning of deterioration of reinforcement and guaranteeing long service life of the structure.

## 1. Introduction

Reinforced concrete structures play a significant role in the construction of infrastructures such as subsea tunnels, urban subways, and high-speed railways and are involved in the lives of millions of people [1]. When reinforced concrete structures are used in a corrosive environment (such as the ocean and saline soil), chloride ion erosion is the main cause of corrosion of steel bars and, ultimately, the deterioration of reinforced concrete structures [2,3]. A useful or practical way to reduce corrosion-induced damage is through prevention, such as real-time monitoring of the Cl^−^ concentration in the environment and concrete.

Traditional methods of detecting chloride ion concentration in concrete structures include drilling concrete at different depths to obtain powder samples and determining the chloride ion content in concrete powder by the potentiometric titration method [4,5]. This technology is usually time-consuming and destructive to the integrity of concrete [6,7]. Furthermore, this technology is unable to continuously monitor the development of chloride ion content in concrete. Differing from the destructive method, non-destructive techniques such as fiber grating and embedded sensors could be used to realize the continuous monitoring of chloride content in concrete [8,9]. Dong et al. [10] used the fiber grating method to measure the concentration of chloride ions in concrete, but it can only obtain the pre-set threshold concentration and cannot be reused. In addition, the optical fiber is susceptible to vibration during the embedding process in concrete, causing the core to break. All-solid-state chloride sensors have chloride-sensitive substances that do not readily decompose in concrete environments, and they are mechanically strong and have stable volume. Compared with fiber grating sensors, embedded sensors such as chloride sensors generally have better chemical stability and simple manufacturing processes [11,12].

The working electrode of the chloride sensor is generally a Ag/AgCl electrode, which has the characteristics of simple preparation and stable performance [13,14]. Since the 1990s, Ag/AgCl electrodes have been widely used in the monitoring of chloride ion content in concrete [15]. Climent-Llorca et al. [16] reported that a Ag/AgCl electrode embedded in a mortar sample maintained good stability over three months. The preparation methods of Ag/AgCl include the physical powder pressing method [17,18], thermal decomposition method, and constant current anode polarization method [19,20]. The Ag/AgCl electrode prepared by the physical powder compaction method is easily powdered and peeled off, so the conductivity of the electrode becomes poor. The product of the thermal decomposition method is impure and the amount of production is difficult to control, resulting in insufficient accuracy of the Ag/AgCl electrode. The Ag/AgCl electrode prepared by the galvanostatic anodic polarization method has better overall performance, but there is a lot of controversy among researchers about the determination of the optimal current density and polarization time during the preparation of the Ag/AgCl electrode. Atkins, C.P. et al. [13] reported that electrodes were anodized in 0.1 mol/L HCl at a current density of 0.4 mA/cm^2^ for 30 to 40 min; Pargar et al. [21] adopted the polarization parameters of 0.5 mA/cm^2^, 1 h; Montemor et al. [22] adopted the polarization parameters of 2 mA/cm^2^, 30 min; Tang et al. [23] prepared a Ag/AgCl chloride ion probe with a current density of 1 mA/cm^2^ in 0.1 mol/L of HCl solution for 30 min.

At present, the chloride sensor based on the electrochemical method usually consists of a working electrode and a reference electrode. The performance of the reference electrode is of vital importance to obtain accurate reliable Cl^−^ content information using the chloride sensor. The types of reference electrodes are saturated calomel electrode, Cu/CuSO_4_ electrode, Hg/HgO electrode, Mn/MnO_2_ reference electrode, etc. [24,25]. When applied to reinforced concrete structures, the monitoring results of external reference electrodes are not reliable. However, the saturated calomel electrode and the Hg/HgO electrode are extremely fragile [26]. The Cu/CuSO_4_ electrode is bulky and the internal solution is easily leached. The above three kinds of electrodes are not suitable to be used as reference electrodes for embedded sensors in concrete. The Mn/MnO_2_ electrode has stable performance and has been used to monitor the chloride ion content in concrete [27,28]. The Mn/MnO_2_ reference electrode prepared by the powder compaction method has higher accuracy and better stability in concrete [29].

In this study, a composite solid chloride sensor consisting of a Ag/AgCl working electrode and Mn/MnO_2_ reference electrode was prepared. The optimal parameters of the Ag/AgCl electrode prepared by the anodic polarization method were studied. The electrochemical performances such as stability, reproducibility, and sensitivity of the composite chloride sensor and single electrode were tested in saturated Ca(OH)_2_ solution and mortar specimens. This work provides technical support for the in situ monitoring of chloride ions in cement mortar.

## 2. Experimental Materials and Methods

### 2.1. Preparation of Ag/AgCl Electrode

**Figure 1:**
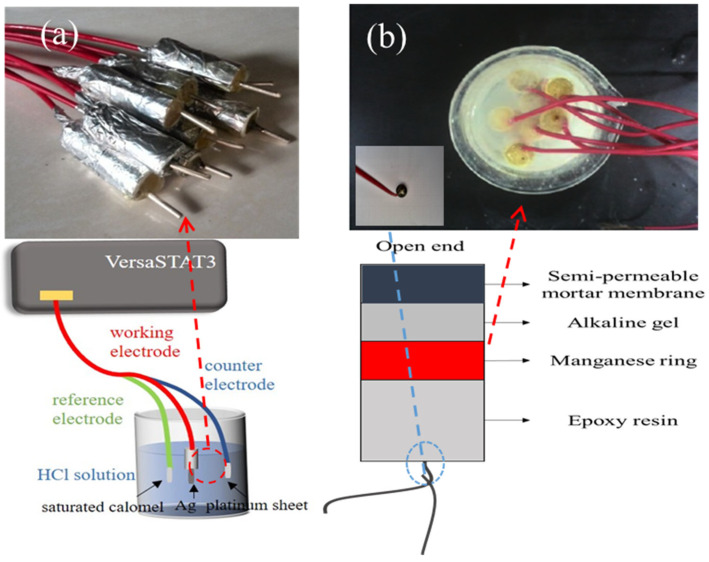
(**a**) Images depicting the preparation of Ag/AgCl electrodes and (**b**) Photographs along with a schematic diagram illustrating the Mn/MnO_2_ reference electrode.

The photos and preparation process of the Ag/AgCl electrode are shown in Figure 1a. One end of the silver wire was tightly welded to the copper wire, and the welded part was sealed with epoxy resin. The final exposed length of the silver wire was 1.5 cm. Then, the silver wire was polished with 600 #, 800 #, and 1000 # sandpaper, immersed in acetone for 10 min to degrease, immersed in 5% nitric acid solution for 1 min, and then washed with anhydrous ethanol and distilled water in sequence.

After completing the above pretreatment of the electrode, the three-electrode system on the Princeton VersaSTAT3 electrochemical workstation was used for galvanostatic polarization, as shown in Figure 1a. The pretreated Ag wire (1.5 cm length) served as the working electrode, a saturated calomel electrode (SCE) was used as the reference electrode, and a platinum sheet electrode served as the counter electrode. The Ag wires were anodized in 0.1 mol/L HCl solution. In this study, the current densities used for anodizing were 0.5 mA/cm^2^, 1 mA/cm^2^, 2 mA/cm^2^, 3 mA/cm^2^, and 4 mA/cm^2^, and the polarization times were 0.5 h, 1 h, 1.5 h, 2 h, 2.5 h, and 3 h. After anodization, the prepared Ag/AgCl electrodes were then placed in 0.1 mol/L KCl solution for activation.

### 2.2. Preparation of Mn/MnO_2_ Reference Electrode

The photos and preparation process of the Mn/MnO_2_ electrode are shown in Figure 1b. The manganese dioxide electrode was prepared according to the research method of Tian et al. [30]. In this study, manganese powder, manganese dioxide powder, carbon powder, and polytetrafluoroethylene as a binder were mixed in a ratio of 1:6:2:1. A high-speed mixer was used to mix the raw materials. After that, the mixture was ground into powder in an agate mortar. Then, a pressure-testing machine was used for loading at a loading speed of 0.2 kN/s. After loading to the target pressure of 40 kN, the load was held for 10 min. A Mn/MnO_2_ manganese ring (d = 12 mm, h = 5 mm) was obtained by demolding. The copper wire was then welded to the surface of the manganese ring and epoxy resin was applied to the weld. The schematic diagram of the Mn/MnO_2_ electrode is shown in Figure 1b. A PVC pipe with an inner diameter of 14 mm was used as the outer shell. The internal filling consisted of 4 layers, which were a semi-permeable mortar membrane (5 mm), alkaline gel (5 mm), manganese ring (5 mm), and epoxy resin (20 mm). Then, the Mn/MnO_2_ reference electrode was placed in a saturated Ca(OH)_2_ solution for activation.

### 2.3. Performance Test of Ag/AgCl Working Electrode

(1) Microscopy analysis

The morphology of the AgCl formed on the surface of Ag was analyzed by using a scanning electron microscope (SEM, FEI-QUANTA250, USA) and an energy dispersive spectrometer (EDS, GENESIS Apollo X incorporated with SEM). The samples were examined under an accelerating voltage of 15 kV in high-vacuum mode. The chemistry of the AgCl layer surface was evaluated through an X-ray diffractometer (XRD, Bruker, D8, Germany).

(2) Response time and calibration of Ag/AgCl electrode

The nature of the response time is the time required for the electrode membrane and ions to establish exchange equilibrium. Response time reflects the sensitivity of the Ag/AgCl electrode to chloride ions and it is an important parameter for the working electrode of a chloride sensor. The prepared Ag/AgCl electrode was put into saturated Ca(OH)_2_ solution with different NaCl concentrations, and the NaCl concentrations were 0.001 mol/L, 0.01 mol/L, 0.1 mol/L, 0.5 mol/L, and 1 mol/L. Then, the open-circuit potential (OCP) of the prepared Ag/AgCl electrode vs. saturated calomel electrode (SCE) was recorded every 30 s. The time when the value of recorded potential is constant is the response time. After each measurement, the electrode should be cleaned with distilled water to prevent the residual solution from affecting the test results.

Calibration of a Ag/AgCl sensor is a necessary step in evaluating its performance when in contact with the external medium [19]. The electrode potentials were tested at a constant temperature of 20 ± 2 °C. Saturated Ca(OH)_2_ solution with different NaCl concentrations was used to calibrate the Ag/AgCl electrode. After calibration, the sensitivity of the Ag/AgCl electrode prepared by other scholars under different polarization parameters was compared to confirm that the Ag/AgCl electrode prepared in this study is reliable.

(3) Reproducibility of Ag/AgCl electrode

The reproducibility of electrodes is one of the important parameters for evaluating electrode performance. The reproducibility of an electrode generally refers to the difference in electrode potential between different electrodes, that is, the degree of reproducibility of the electrode potential. In this paper, four Ag/AgCl electrodes prepared in the same batch were put into saturated Ca(OH)_2_ solution with different NaCl concentrations to test the potential.

(4) Stability of Ag/AgCl electrode

The stability of an electrode usually refers to the measurement of potential over a period of time, and the performance of the electrode potential is stable within the allowable range of error. The Ag/AgCl electrodes were placed in saturated Ca(OH)_2_ solution with different NaCl concentrations. The potential between the working electrode and the saturated calomel electrode was measured. The potential value was continuously recorded over 45 days. Each day, the solution was changed to ensure a constant pH of the solution during the test. To prevent the water in the solution from evaporating, the container was sealed.

(5) Anti-polarization capability of Ag/AgCl electrode

When an electric current passes through the electrode, the phenomenon in which the electrode potential deviates from the equilibrium potential is called electrode polarization [31]. Electrochemical impedance spectroscopy (EIS) was used to evaluate the impedance characteristics of the electrode. The frequency range of EIS was 10^5^–10^−2^ Hz, and the AC amplitude was 10 mV. The Tafel curve was used to find the exchange current density and reversibility of the electrode. The potential sweep interval was ±300 mV of the open circuit potential, and the sweep speed was 0.5 mV/s. The test solution used in the experiment was 0.1 mol/L NaCl aqueous solution.

### 2.4. Performance Test of Mn/MnO_2_ Reference Electrode

(1) Response time of the reference electrode

The Mn/MnO_2_ electrode and saturated calomel electrode were immersed in saturated Ca(OH)_2_ solution with different NaCl concentrations. The time required for the measurement potential in each solution to reach a stable state was obtained, which is the response time of the Mn/MnO_2_ electrode.

(2) Stability of the reference electrode

The prepared Mn/MnO_2_ reference electrode was immersed in a saturated Ca(OH)_2_ solution with 0.1 mol/L chloride to test its potential relative to the saturated calomel electrode. By continuously recording the electrode potential for 60 days, the stability of the reference electrode was analyzed.

### 2.5. Performance Test of Solid Chloride Sensor

(1) Response time and Nernst response in saturated Ca(OH)_2_ solution with different NaCl concentrations

The Ag/AgCl working electrode and the Mn/MnO_2_ reference electrode constitute a chloride sensor. The response time of the chloride sensor refers to the time it takes for the electrode potential to reach the equilibrium potential when the sensor is placed in the solution. In five kinds of saturated Ca(OH)_2_ solutions with different chloride ion concentrations, the potential and response time of the chloride sensor were tested using a multimeter and a stopwatch. Three stable times were taken in the same solution, and the average value was taken as the final response time. The sensor was washed with distilled water when the test was completed.

At the same time, the potential of the sensor under different chloride ion concentrations was also measured in the experiment. By analyzing the correlation between potential and chloride ion concentration, we confirmed the Nernst equation for the chloride sensor and performed a calibration of the sensor.

(2) Stability in saturated Ca(OH)_2_ solution with different NaCl concentrations

The stability of the chloride sensor refers to the electrode potential being stabilized within the allowable range of error during the period of potential measurement. A three-electrode system of the Princeton VersaSTAT3 electrochemical workstation was used to test the change in sensor potential with time in saturated Ca(OH)_2_ solution with different NaCl concentrations, and the potential was continuously measured for 38 days. The Ag/AgCl electrode served as the working electrode, the Mn/MnO_2_ electrode served as the reference electrode, and the platinum electrode served as the auxiliary electrode.

(3) Reproducibility in saturated Ca(OH)_2_ solution with different NaCl concentrations

The four chloride sensors were placed in a saturated Ca(OH)_2_ solution with a chloride ion concentration of 0.001 mol/L, and the potentials of all electrodes were tested at 7, 14, 21, and 28 days. By comparing the difference in potential of different electrodes in the same solution, the reproducibility of the sensor was obtained.

(4) Stability in mortar

**Figure 2:**
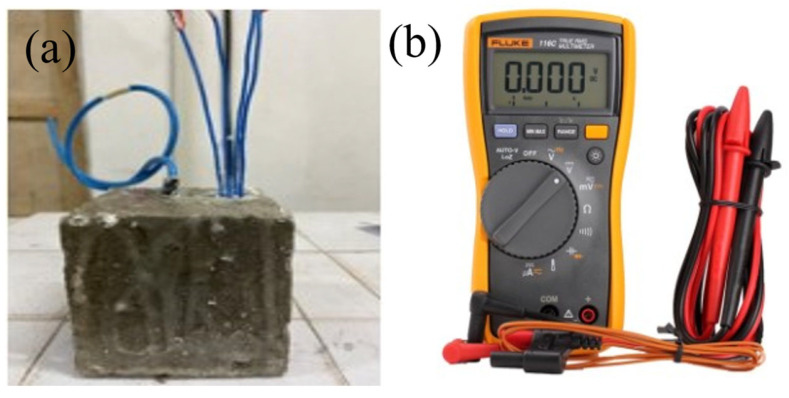
Photos of (**a**) the mortar specimens with the chloride sensors and (**b**) equipment measuring sensor potential.

The chloride sensor was buried in mortar with a water–cement ratio of 0.6 (as shown in Figure 2), and its performance in the mortar was tested. During the mixing state, NaCl was dissolved into mixing water to provide Cl^−^ and the NaCl content (relative to the mass of cement) is shown in Table 1. Prior to casting the mortar, we initially secured the chloride sensor at the center of the mortar mold. After the pouring of the mortar sample was completed, the potential of the chloride sensor was measured. Through the relationship curve between sensor potential and time, the stability performance of the sensor was analyzed.

(5) Nernst response in mortar

The amount of sodium chloride added to the mortar is known, as shown in Table 1, but during the cement hydration process, part of the chloride ion will be physically adsorbed and chemically combined with the hydration product, which changes the chloride ion content in the pore solution. Therefore, to establish the Nernst equation of the chloride sensor, the chloride ion content inside the mortar needs to be measured. After the potential of the chloride sensor was stabilized, the specimens of mortar were cut, and the powder at the position of the chloride sensor was drilled with a drill. The content of chloride ions in all mortars was measured by chemical titration. According to the relationship between the potential of the chloride sensor and the chloride ion content, the Nernst equation was obtained and the sensor was calibrated.

## 3. Results and Discussion

### 3.1. Performance of Working Electrode

#### 3.1.1. Transient Analysis of the Formation Process of AgCl Film

**Figure 3:**
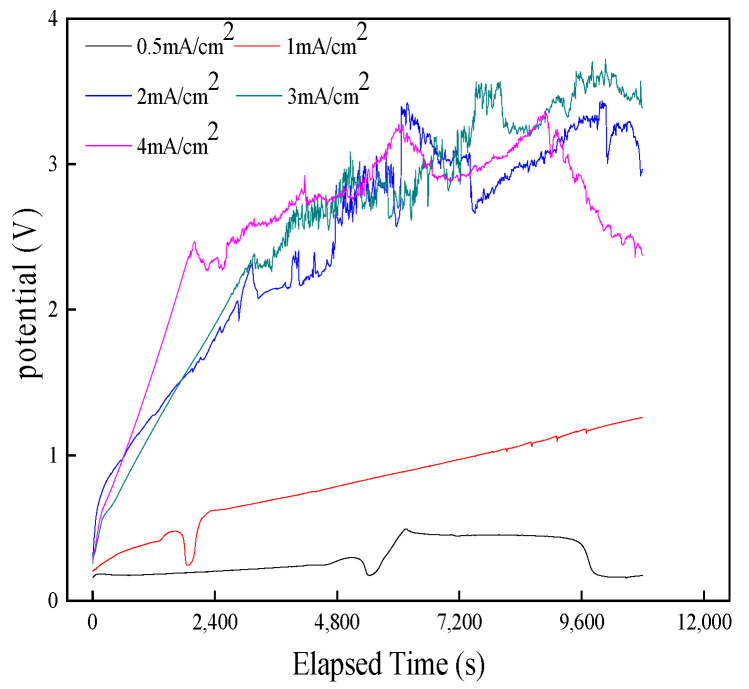
Potential–time curve of Ag/AgCl electrode under different current densities.

Under different current densities, the potential–time curve of the Ag/AgCl electrode during electrolysis is shown in Figure 3. With the increase in the polarization time, the potential value of the electrode under different current densities showed an upward trend, indicating that the longer the polarization time, the greater the thickness of the film and the greater the electrode resistance. Therefore, the electrochemical workstation must output a higher potential at a constant current to resist increasing resistance. The greater the current density, the faster the oxide film is generated and the faster the potential increases.

When the current density was 0.5 mA/cm^2^, the potential suddenly dropped around 9600 s, and then the potential increased again. The reason may be that when the polarization time was about 9600 s, the oxide film of the electrode cracked or fell off so that the Ag wire was exposed, which increased conductivity and decreased potential. After a while, the exposed Ag wire was oxidized again, and the potential showed an upward trend. When the energized current density was 1.0 mA/cm^2^, the trend of its growth phase was roughly the same as at the current density of 0.5 mA/cm^2^. The difference is that the slow growth period in the early period is shorter, and the steady growth period in the later period is longer. When the current density is 2.0, 3.0, and 4.0 mA/cm^2^, the potential growth trend of the electrode is similar, both increasing rapidly and then fluctuating frequently. This indicates that the AgCl film formed under this energizing current density has poor compactness, adhesion, and firmness.

#### 3.1.2. Micromorphology of Ag/AgCl Electrode

**Figure 4:**
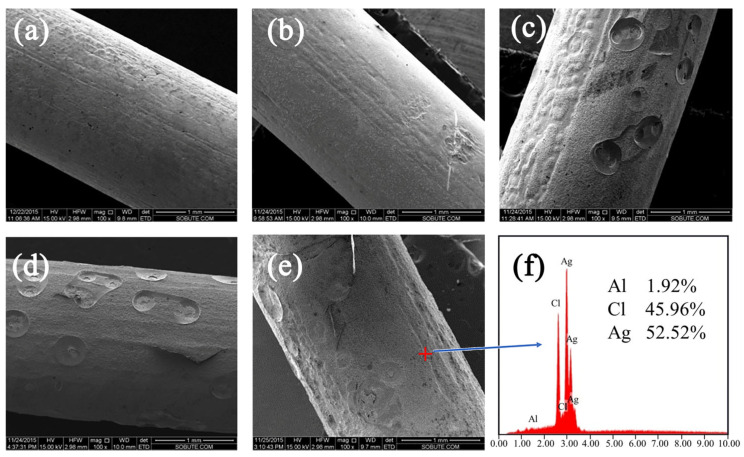
SEM (100×) morphology of the Ag/AgCl electrode under different current densities: (**a**) 0.5 mA/cm^2^, (**b**) 1 mA/cm^2^, (**c**) 2 mA/cm^2^, (**d**) 3 mA/cm^2^, (**e**) 4 mA/cm^2^, and (**f**) EDS spectrum of Ag/AgCl electrode under current density of 4 mA/cm^2^ at polarization time of 2.5 h.

**Figure 5:**
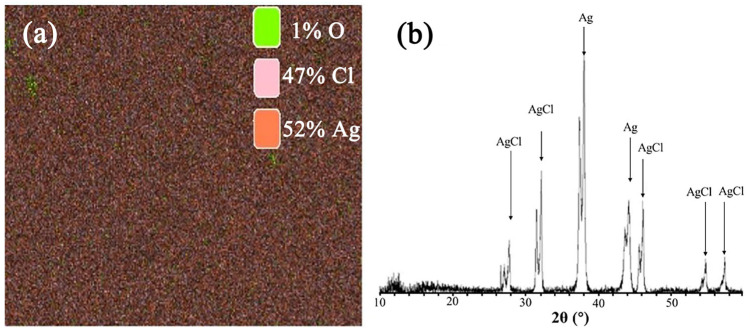
(**a**) EDX mapping of AgCl electrode and (**b**) XRD result of electrolytic products (0.5 mA/cm^2^, 2.5 h).

As shown in Figure 4, when the polarization time is constant, the surface of the Ag/AgCl electrode becomes rougher with the increase in current density. When the current density is higher than 2 mA/cm^2^, pits and bumps appeared on the surface of the Ag/AgCl electrode, and it was loose with low adhesion. The reason is as follows: the process of anodization forms a continuous non-porous thin film layer on the Ag substrate first and then continues to electrolytically generate a porous thin film layer. However, due to the high current density, silver ions are formed on the surface of the Ag/AgCl electrode after the loss of electrons, which reduces the conductivity of the electrode. At this time, the metallic silver of the portion is no longer electrolyzed, and the hydroxide in the solution is electrolyzed, thereby generating oxygen which concentrates on the surface of the Ag/AgCl electrode. The oxygen atoms generated by electrolysis combine with silver ions to form a silver oxide, which adheres to the surface of the Ag/AgCl electrode. Another portion of the oxygen atoms continue to react to form oxygen, which adheres to the surface of the electrode, and the part in the pit is caused by bubbles. As shown in Figure 4f, EDS confirmed the presence of microtraces of the O element under electrolysis conditions. The aluminum element detected in the EDS result is an impurity that arises during the electrode electrodeposition process. As shown in Figure 5, XRD results showed that the ratio of the atomic weight of silver to the atomic weight of chlorine was close to 1:1, and no silver oxide was found. The reason is that XRD analysis involved a large area scan, and tiny amounts could not be found. Compared to the XRD, the composition results determined by EDS are more accurate due to the point scan mode. Thus, the result of XRD is not representative. Therefore, the presence of an O element is to be expected. Furthermore, under the influence of a higher current density, the growth rate of the silver matrix film layer accelerated, resulting in a thicker film that exhibited reduced conductivity, and silver ceased to be electrolyzed into silver ions. The growth rates of the various layers were not synchronized, resulting in some localized film layers forming faster than in the nearby regions, causing a bump. In these cases, the resulting electrode film layer is less compact, easily peels off, and has poor adhesion.

**Figure 6:**
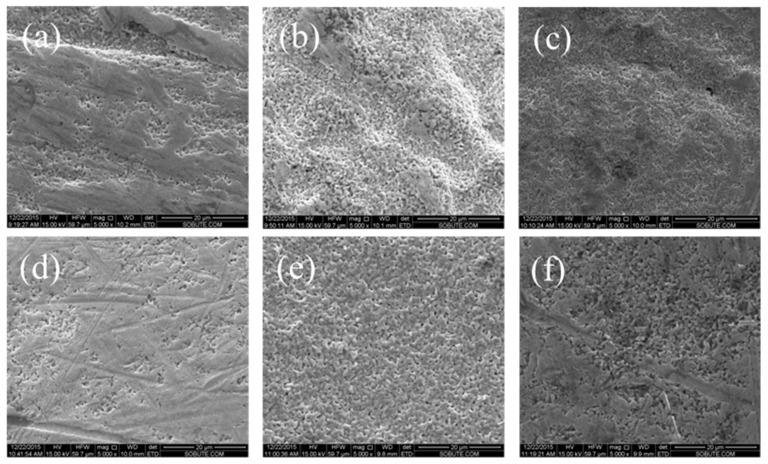
At current density of 0.5 mA/cm^2^, SEM (5000×) morphology of Ag/AgCl electrode under different polarization times: (**a**) 0.5 h, (**b**) 1 h, (**c**) 1.5 h, (**d**) 2 h, (**e**) 2.5 h, and (**f**) 3 h.

When current density was constant and less than 1 mA/cm^2^, the electrode surface became progressively denser with an increase in polarization time. When the polarization time is too long, the oxide film on the electrode surface will become uneven in thickness and the pore distribution will be irregular, as shown in Figure 6. After anodization for 0.5 h, the surface of the AgCl layer was irregularly shaped, with complex patterns of AgCl particles and a porous structure. As the anodization time further increased to 1 h and 1.5 h, the surface of the AgCl layer was irregular and the small AgCl particles formed were stacked on top of each other. After anodization for 2 h, the surface of the AgCl layer was highly uneven. When the polarization time was 2.5 h, the small particles of AgCl were arranged regularly and the pores formed were evenly distributed. After anodization for 3 h, the surface of the AgCl layer is highly distorted and the AgCl particles are disorderly stacked. The irregularity of the interlayer cavities can be easily observed. From the obtained results, it can be concluded that the complexity of the layer is respective to the anodization time. If the polarization time is too long, the adhesion of the AgCl layer on the Ag substrate will decrease, forming a complex multilayer structure, which will affect the performance of the AgCl electrode. Under different polarization times, the change in electrode surface is consistent with the change in electrochemical response in Figure 3. Therefore, the electrode prepared with a polarization time of 2.5 h and a current density of 0.5 mA/cm^2^ is relatively more stable.

#### 3.1.3. Response Time of Ag/AgCl Electrode

**Figure 7:**
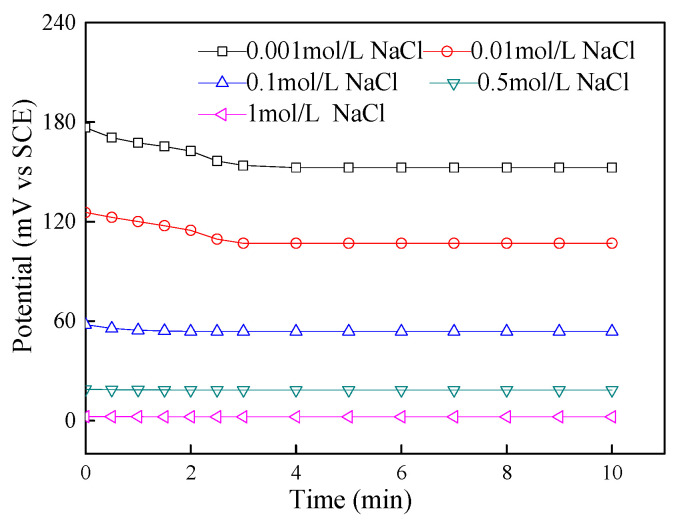
Potential–time curve of Ag/AgCl electrode in saturated Ca(OH)_2_ solution with different NaCl concentrations.

As shown in Figure 7, the response time of the Ag/AgCl electrode increased as the chloride ion concentration decreased. The response time of the Ag/AgCl electrode in saturated Ca(OH)_2_ solution with different NaCl concentrations was 30 s to 3 min. In a low-concentration chloride solution, the activity of chloride ions is lower and the response time of the electrode is longer. In contrast, in a chloride solution with a high concentration, the more active the ions, the shorter the response time of the electrode is. The response time of the electrode is related to the concentration of chloride ions, and the response time is very short at high concentrations, which helps the application of electrodes to monitor chloride ions.

#### 3.1.4. Nernst Response of Ag/AgCl Electrode

**Figure 8:**
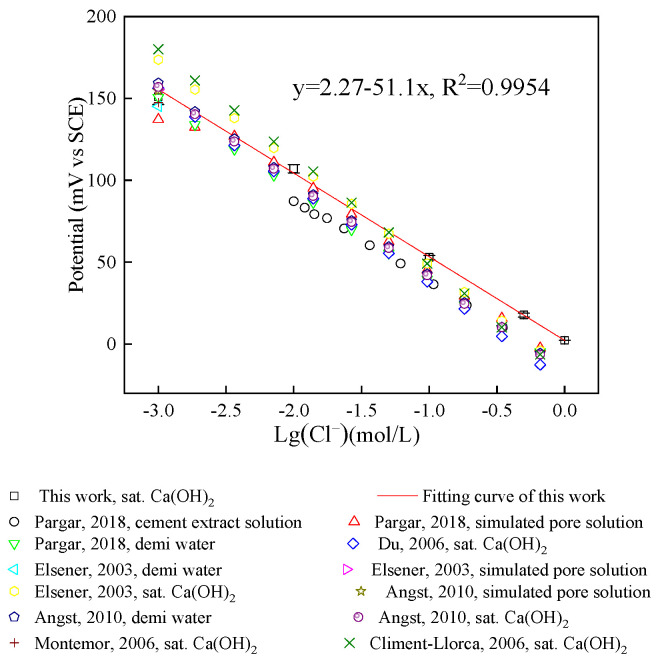
The sensitivity of Ag/AgCl electrode potential to chloride ion content in this paper and previously published literature.

Figure 8 is the relationship between the chloride ion content and the potential of the Ag/AgCl electrode in saturated Ca(OH)_2_ solution with different NaCl concentrations, together with values reported by other researchers. The potential of the Ag/AgCl electrode is linearly related to the logarithm of the chloride ion concentration, the correlation coefficient (R^2^) is 0.99, and the sensitivity is 51.1 mV/decade. The deviation of the result from the theoretical value may be related to the activity coefficient of chloride ions [31]. The research result of Jin et al. [11] on the sensitivity of Ag/AgCl electrodes to chloride ions in solution with pH = 13.5 was 53.66 mV/decade. Table 2 summarizes the results of other scholars on the calibration curve. The slope of the calibration curve varies from −50 mV/decade to −61.72 mV/decade. Although the slope of the calibration curve of the Ag/AgCl electrode prepared in this study is basically the same as that of other scholars, it is different from the theoretical slope. The theoretical Nernst slope is based on the thermodynamic equilibrium and spontaneous reaction of the sensor surface under standard conditions, and the experimental Nernst slope is derived under the relevant experimental conditions. Changes in the test medium (such as changes in alkalinity or ionic strength), as well as the presence of average activity coefficients and liquid junction potentials [32], result in different slopes of the calibration curve. Therefore, the difference between the theoretical and experimental calibration curve slopes is reasonable.

#### 3.1.5. Stability and Reproducibility of Ag/AgCl Electrode

**Figure 9:**
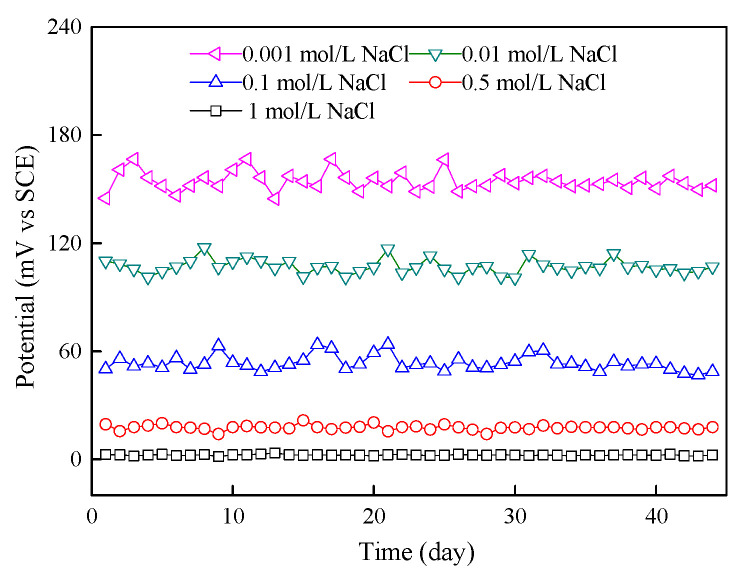
Potential–time curve of Ag/AgCl electrode in saturated Ca(OH)_2_ solution with different NaCl concentrations.

Figure 9 shows the change in potential of the Ag/AgCl electrode with time in saturated Ca(OH)_2_ solution with different NaCl concentrations. In a solution with a high chloride concentration, such as 0.5 mol/L or 1 mol/L, the chloride ion activity is high, and the potential of the Ag/AgCl electrode stabilized quickly and remained stable during the test. In a solution with a low chloride ion concentration, such as 0.001 mol/L, the chloride ion activity is low, the electrode potential fluctuates above and below the equilibrium potential, and it takes a long time to stabilize. The same results are reported by Angst et al. [31]. That is because the establishment of ion balance in the solution and the stabilization of the Ag/AgCl electrode require time. In addition, under an environment of low chlorine and high alkalinity, the Ag/AgCl electrode is affected by hydroxide ions and the formed AgCl may be converted into Ag_2_O in a small amount. Furthermore, at the beginning of the test, the potential of the Ag/AgCl electrode in each solution fluctuated greatly, but at the end of the test, the potential in all saturated Ca(OH)_2_ solutions with different NaCl concentrations fluctuated within 5 mV.

**Figure 10:**
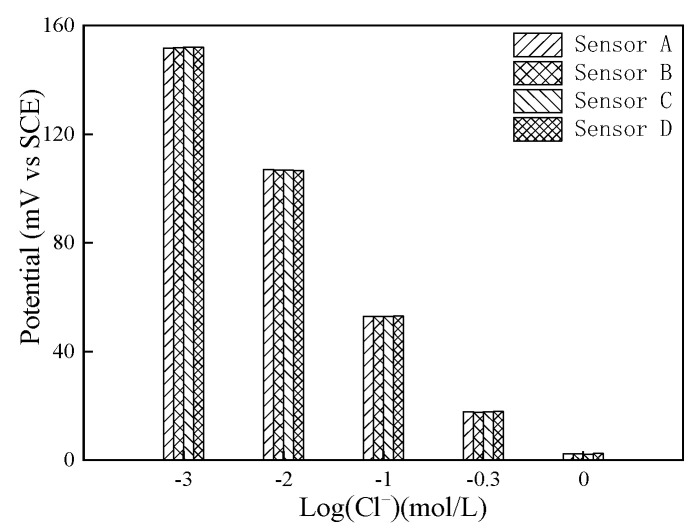
Potential of different electrodes in saturated Ca(OH)_2_ solution with different NaCl concentrations.

Figure 10 shows that the potential values of the four electrodes in the same chloride ion concentration simulation solution were nearly identical, the potential deviation was small, and the difference in potential of the Ag/AgCl electrode was between 0.2 and 0.5 mV. The prepared Ag/AgCl electrode has good reproducibility.

#### 3.1.6. Anti-Polarization Capability of Ag/AgCl Electrode

**Figure 11:**
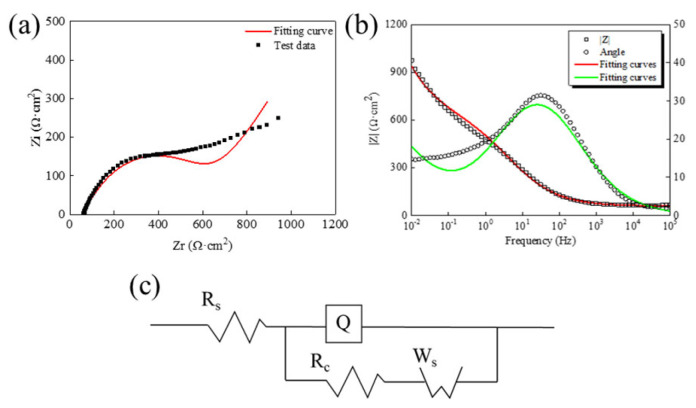
In the 0.1 mol/L NaCl solution, (**a**) Equivalent circuit diagram, Nyquist plot and (**b**) Bode plot, and (**c**) Equivalent circuit diagram.

The electrochemical impedance spectroscopy of the Ag/AgCl electrode is fitted by ZSimpWin software 3. 30 according to R_s_ (Q(R_c_W_s_)) and the equivalent circuit diagram in Figure 11a, and the fitting results are shown in Table 3, where Rs is the solution resistance, R_c_ is the charge transfer resistance of the electrode reaction, and W_s_ is the Warburg diffusion resistance of particles in the AgCl solid electrolyte. In this model, the constant phase angle element Q is used instead of the pure capacitor element to solve the non-ideal capacitive response.

In Figure 11a, the semi-circular ring in the high-frequency region is related to the charge transfer resistance R_c_ and the film capacitance C_dl_. The R_c_ (542.2 Ω·cm^2^) of the Ag/AgCl electrode is much smaller than that of Pargar et al.’s result (22 kΩ·cm^2^) [21] in a 0.125 mol/L sodium chloride solution. Pargar et al. pointed out that the smaller the R_c_, the less the electrode is disturbed by the current, which proved that the prepared Ag/AgCl electrode in this paper has better anti-polarization capability. Compared with the rolled Ag/AgCl electrode C_dl_ (1.62 × 10^−4^ F·cm^−2^) prepared by Gao et al. [34], the prepared Ag/AgCl electrode in this paper has a smaller C_dl_ and a relatively denser surface film. In the low-frequency region, a straight line with an angle close to 45° with the real axis appears, which is consistent with the characteristics of Warburg impedance in electrochemistry [35].

**Figure 12:**
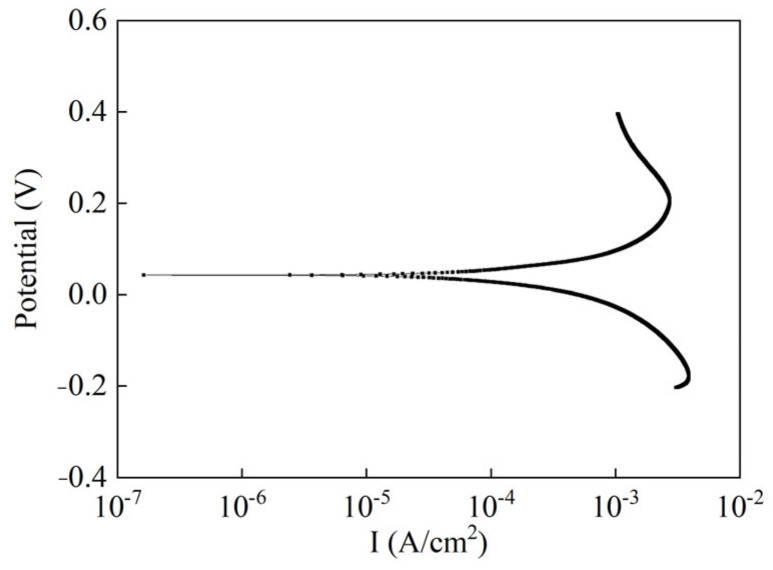
Tafel curve of Ag/AgCl electrode in 0.1 mol/L NaCl solution.

The Tafel curve of the Ag/AgCl electrode is shown in Figure 12. Compared with the electrode exchange current density (3.77 × 10^−4^ A/cm^2^) by Gao et al. [34], the exchange current density of the electrode in this paper is 4.35 × 10^−4^ A/cm^2^. The polarization curve reflects the dynamic process of the electron transfer step on the electrode surface. The greater the exchange current density, the faster the electron exchange rate between Ag and Ag^+^, that is, the stronger the electrode’s anti-polarization ability.

### 3.2. Stability of Potential of Mn/MnO_2_ Reference Electrode

**Figure 13:**
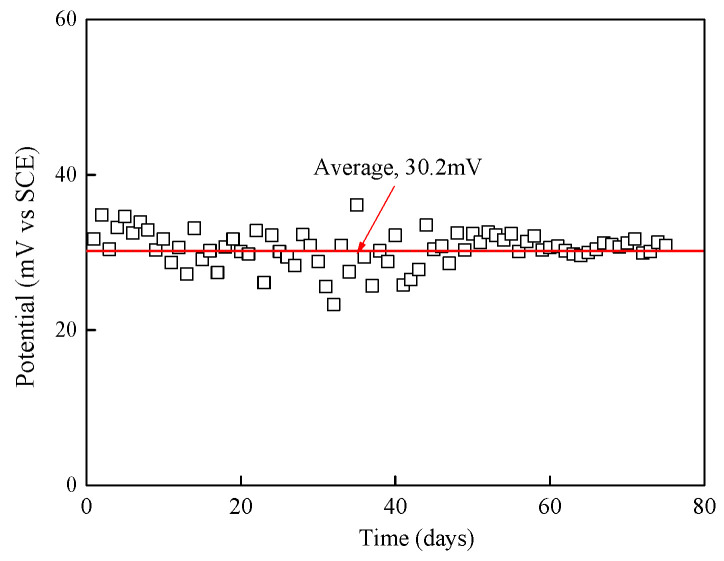
Potential–time curve for Mn/MnO_2_ electrodes.

In order to test the stability Mn/MnO_2_ electrodes, the potential change of the Mn/MnO_2_ electrode was measured using a commercial saturated calomel electrode (SCE) as the reference electrode. The relationship between the potential of the Mn/MnO_2_ electrode and time is shown in Figure 13. The potential of the Mn/MnO_2_ electrode fluctuated greatly in the initial stage of the test. With the increase in the test time, the potential of the Mn/MnO_2_ electrode gradually stabilized and finally reached the equilibrium potential of 30.2 mV.

At the beginning of the test, due to insufficient activation time, MnO_2_ was in the activation period. The β-MnO_2_ in the active material of the Mn/MnO_2_ reference electrode can only maintain a homogeneous state for a short time and is converted into the γ-MnOOH phase during activation, as shown in Equation (1). The conversion of β-MnO_2_ to γ-MnOOH is random, and when the MnO_2_-MnOOH system is in the polarization or charge–discharge process, the ratio of MnO_2_ to γ-MnOOH of the surface of the electrode is changed, and the open circuit potential also changes accordingly.

(1) $${\mathrm{MnO}}_{2}+H^{+}+e\to \mathrm{MnOOH}$$

With the extension of the activation time, the MnO_2_, Mn, and MnO_X_ in the sample were analyzed by the FeSO_4_ method, and X was 1.5, inferring that the oxide type was Mn_2_O_3_. In addition, MnO_2_, Mn, and Mn_2_O_3_ establish a multiphase balance based on thermodynamic principles, and the performance of the reference electrode will be relatively stable, reflected in the fluctuation of the potential within the allowable range of error.

### 3.3. Performance of Chloride Sensor

#### 3.3.1. Response Time of Chloride Sensor in Saturated Ca(OH)_2_ Solution

Table 4 shows the response time of chloride sensors in saturated Ca(OH)_2_ solution with different NaCl concentrations. As the concentration of chloride ion increases, the response time becomes shorter. Compared with the test result with the SCE as the reference electrode in Figure 7, the chloride sensor has a shorter response time (30 s) in a low-concentration chloride ion solution (0.001 mol/L). In the solution with a high chloride ion concentration, the ion activity is relatively high, and the response time of the chloride sensor is the same as that when the SCE is the reference electrode. It is suggested that in the actual application of this kind of chloride sensor, the data should be read after 30 s.

#### 3.3.2. Nernst Response of Chloride Sensor in Saturated Ca(OH)_2_ Solution

**Figure 14:**
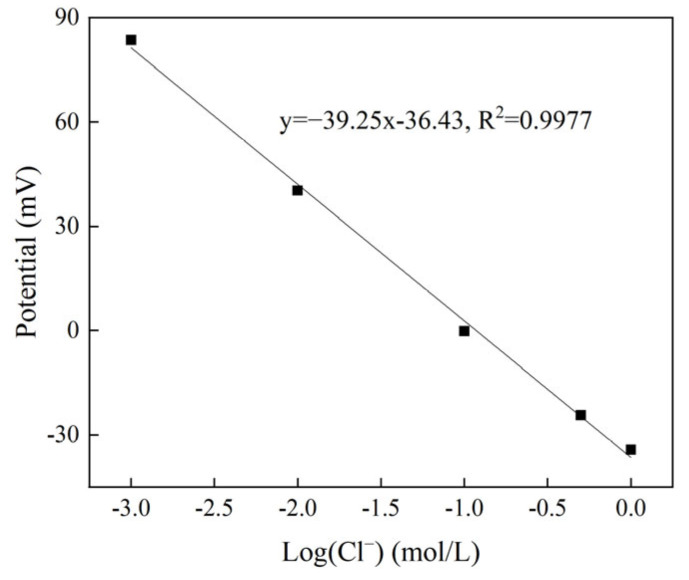
Potential of chloride sensor as a function of chloride ion content in synthetic concrete pore solution.

According to the Nernst equation, the potential value of the Ag/AgCl working electrode has a linear relationship with the negative logarithm of the chloride ion concentration in the solution. Figure 14 shows the potential of the chloride sensor in a saturated Ca(OH)_2_ solution with different NaCl concentrations. As the logarithm of the chloride ion concentration increases, the potential of the chloride sensor decreases linearly with a linear correlation coefficient of 0.99 and a slope of 39.25 mV/decade. At the same time, in the low-concentration region (0.001 to 0.01 mol/L), the potential of the chloride sensor was slightly different from the calibration curve, which may be due to the interference of OH^−^ [36], and this deviation could be attributed to the formation of AgOH on the AgCl layer [33]. Compared with the test result of the SCE as the reference electrode, the slope of the calibration curve is lower but it still has a good Nernst response.

#### 3.3.3. Stability and Reproducibility of Chloride Sensor in Saturated Ca(OH)_2_ Solution

**Figure 15:**
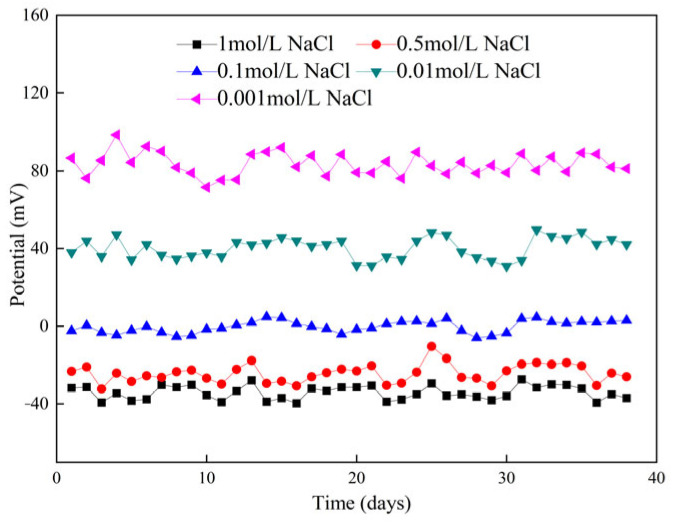
Potential–time curve of chloride sensor in saturated Ca(OH)_2_ solution with different NaCl concentrations.

The change in potential of the Ag/AgCl-Mn/MnO_2_ chloride sensor in chloride-contaminated saturated Ca(OH)_2_ with time is shown in Figure 15. With the increase in time, the potential of the chloride sensor gradually stabilized, the potential fluctuation of the chloride sensor in solution with a low concentration of chloride ion was large, and it took a long time to stabilize. The reason is that the chloride sensor is selective to chloride ions, and in solution with a low concentration of chloride ions, chloride ion activity is low and the working electrode is affected by hydroxide ions, which causes a small amount of Ag_2_O to be generated on the electrode surface. In solution with a high concentration of chloride ions, the chloride sensor is more sensitive to chloride ions and no Ag_2_O is generated on the surface of the working electrode. However, it still takes some time for the electrode to establish ion balance and solution stability.

Therefore, the chloride sensor prepared by the method mentioned in this paper must be activated for more than 35 days before application. The activation solution can be a saturated Ca(OH)_2_ solution with a chloride concentration of 0.001 mol/L.

**Figure 16:**
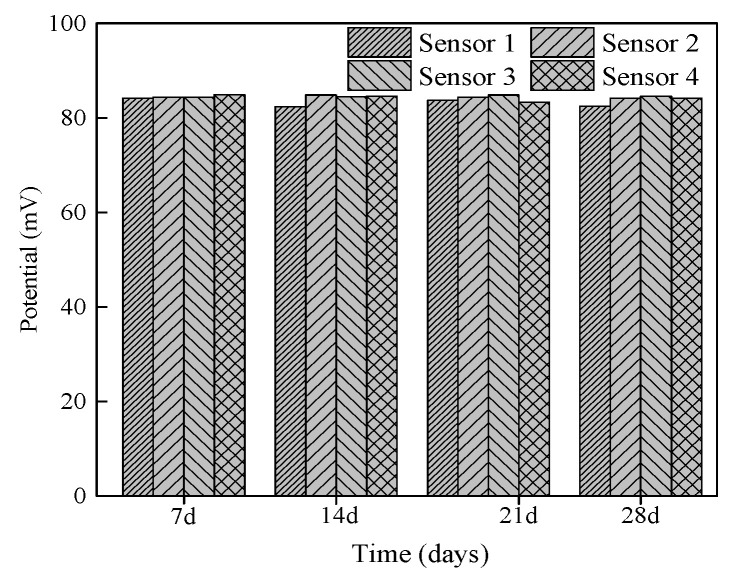
Potentials of different chloride sensors in saturated Ca(OH)_2_ solution with chloride concentration of 0.001 mol/L.

The potentials of four chloride sensors in saturated Ca(OH)_2_ solution with a chloride concentration of 0.001 mol/L at the age of 7 days, 14 days, 21 days, and 28 days are shown in Figure 16. The potentials of different sensors at the same test time were almost the same, and the potentials measured by the same sensor at different test times were the same. The difference in potential between the different sensors is due to errors in the preparation of the Ag/AgCl working electrode and Mn/MnO_2_ reference electrode. It shows that the prepared chloride sensor has good reproducibility and can be prepared in batches.

#### 3.3.4. Calibration of Chloride Sensor in Mortar

**Figure 17:**
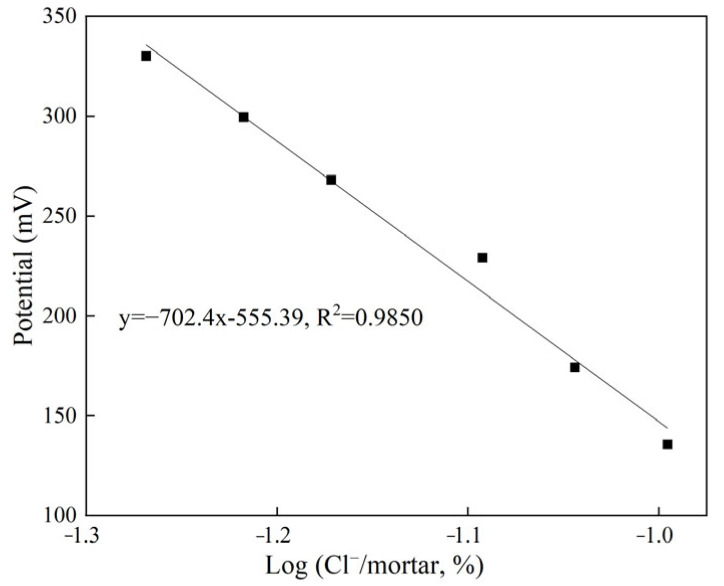
Nernst response of chloride sensor in mortar.

Since the chloride ion content in the mortar pore solution changes during the cement hydration process, if the Nernst equation of the chloride sensor is to be established, the chloride ion content inside the mortar needs to be measured. The relationship between the potential and the negative logarithm of the measured chloride ion content is shown in Figure 17. Obviously, the potential of the chloride sensor is linearly related to the ratio of chloride ion to mortar mass, which conforms to the Nernst equation. This fully shows that the prepared chloride sensor can be used to test the chloride ion content in cement-based materials in situ.

#### 3.3.5. Stability of Chloride Sensor in Mortar

**Figure 18:**
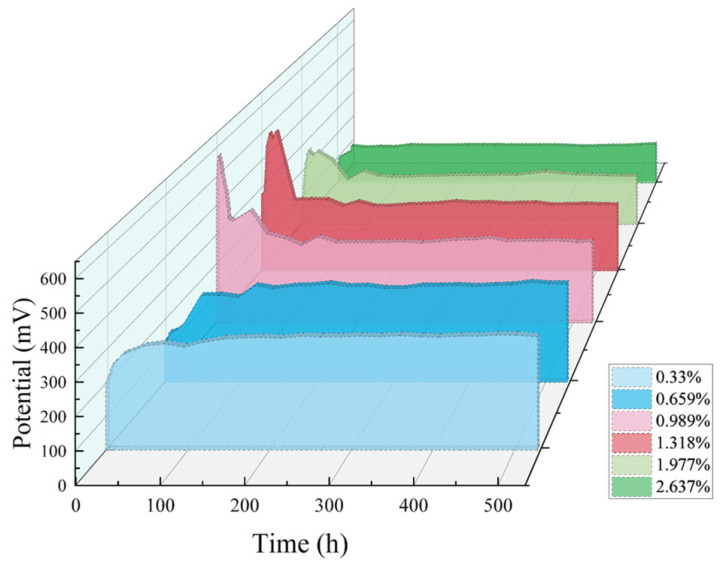
Potential–time curve of chloride sensor in the mortar mixed with different contents of NaCl.

The potential of the chloride sensor in mortars with different chloride ion contents changed with time as shown in Figure 18. Clearly, in the early stage of the mortar sample, the potential of all chloride sensors fluctuates greatly, first increasing and then decreasing. However, after the mortar hardened, the chloride ion concentration and pH value in the pore solution of mortar were basically stable, and the potential of the chloride sensor gradually stabilized, indicating its excellent stability. The reason is as follows: chloride ions can not only physically be adsorbed to the surface of the hydrated calcium silicate product in the mortar but also chemically react with tricalcium aluminate (C_3_A, 3CaO·Al_2_O_3_) and tetracalcium aluminoferrite (C_4_AF, 4CaO·Al_2_O_3_·Fe_2_O_3_) to form Friedel’s salt (3CaO·Al_2_O_3_·CaCl_2_·10H_2_O) and its analogues [37,38]. This will cause the free chloride ion content in the pore solution to decrease, resulting in a decrease in potential.

Given the good performance of the chloride sensor in solutions and mortar specimens, it has great potential for non-destructive monitoring of the chloride content in concrete members. When the sensor is used in concrete it can be tied to reinforcement bars at different depths to detect the depth of chloride ion penetration. The robust electrodeposition layer and the outer protective tube can ensure the integrity and effectiveness of the sensor during the casting and service of concrete members. Furthermore, it should be noted that the interior of concrete is a very complex environment, and there remain some challenges for using the embedded sensor in concrete. Firstly, the influence of the saturation of concrete on the performance should be carefully studied. Secondly, the long-term effectiveness of the chloride sensor needs to be further investigated to match the service life of concrete structures.

## 4. Conclusions

This paper studies the electrochemical performance of the Ag/AgCl working electrode, Mn/MnO_2_ reference electrode, and the composite chloride sensor in saturated Ca(OH)_2_ solution with different NaCl concentrations and mortar. The main conclusions are as follows:

(1) The Ag/AgCl electrode was prepared by the constant current anodic polarization method with different parameters. The film layer generated on the electrode surface becomes looser when a larger current density is applied. The optimal preparation condition for the Ag/AgCl selective electrode is as follows: current density of 0.5 mA/cm^2^ and polarization time of 2.5 h.

(2) The Ag/AgCl electrode potential has a linear relationship with the logarithm of the chloride ion concentration in the solution. The Ag/AgCl electrode also has good electrochemical characteristics such as stability and reproducibility. Ions such as SO_4_^2−^ and Mg^2+^ negatively influence the Ag/AgCl electrode potential. The Mn/MnO_2_ electrode potential has good stability and a small potential fluctuation of 1~3 mV after an activation period of 60 days.

(3) The chloride sensor is prepared by combining the Ag/AgCl working electrode and the Mn/MnO_2_ reference electrode. In saturated Ca(OH)_2_ solution with different NaCl concentrations, the sensor has a short response period (less than 30 s), good stability and reproducibility, and a linear relationship between the potential and the logarithm of the chloride ion concentration (slope is 51.1 mV/decade). In mortar specimens, the sensor shows good stability. Furthermore, there exists a linear relation between sensor potential and the mass fraction of chloride ions in mortar. Thus, in using this chloride sensor for chloride monitoring, the potential value of the sensor can be converted to the free chloride content of the mortar samples.

## Figures and Tables

**Table 1:** The NaCl content relative to the mass of cement.

Cl^−^/cement, %	0.2	0.4	0.6	0.8	1.2	1.6
NaCl/cement, %	0.330	0.659	0.989	1.318	1.977	2.637

**Table 2:** The statistical analysis of the slope of the calibration curve of Ag/AgCl electrode in the simulated pore solution, cement extract, and deionized water.

Solution	Slope						
	This Paper	Ref. [31]	Ref. [16]	Ref. [19]	Ref. [22]	Ref. [33]	Ref. [12]
Saturated Ca(OH)_2_	−51.10	−57.60	−61.72	-	−53.46	−59.00	−56.02
CE	-	-	-	−49.73	-	-	-
SPS	-	−57.80	-	−58.11	-	−54.00	-
DW	-	−58.30	-	−55.39	-	−50.00	-
Theoretical value	−59.16						

**Table 3:** Fitting results of EIS curve of Ag/AgCl electrode in 0.1 mol/L NaCl solution.

R_s_ (Ω·cm^2^)	Q-Y (F·cm^−2^)	Q-n	R_c_ (Ω·cm^2^)	W_s_-Y (F·cm^−2^)
61.43	2.495 × 10^−4^	0.599	542.2	8.159 × 10^−3^

**Table 4:** Response time of chloride sensor in chloride-contaminated Ca(OH)_2_ solution.

Concentration of Cl^−^ (mol/L)	1	0.5	0.1	0.01	0.001
Response time (s)	5	8	10	24	30

## Data Availability

Data are contained within the article.
